# Comparison of the Effects of Beclomethasone Dipropionate and Budesonide in the Treatment of Children with Mild, Persistent Asthma

**DOI:** 10.7759/cureus.17943

**Published:** 2021-09-13

**Authors:** Ibrahim Bukhari, Muhammad Ashfaq, Bader-u- Nisa, Aijaz Ahmed, Hira Waseem, Mehrunnisa Yasir

**Affiliations:** 1 Pediatrics, Sindh Government Hospital, Karachi, PAK; 2 Pediatric Medicine Ward 1, National Institute of Child Health, Karachi, PAK; 3 Pediatric Medical Unit 3, National Institute of Child Health, Karachi, PAK

**Keywords:** asthma, beclomethasone dipropionate, budesonide, peak expiratory ﬂow, inhaled drug

## Abstract

Objective

To compare the mean change in peak expiratory ﬂow values in children receiving inhaled beclomethasone dipropionate versus inhaled budesonide in the treatment of mild persistent asthma.

Method

The medical records of 60 patients from the outpatient department (OPD)/emergency room (ER), National Institute of Child Health, Karachi, who received beclomethasone dipropionate (BDP) 200 µg one puff and budesonide (BUD) 200 µg one puff twice a day for treatment of mild persistent asthma from March 10, 2020, to August 10, 2020, were explored.

Results

The mean age of children was 10.56 ± 3.01 years in the BUD group and 10.05 ± 3.54 years in the BDP group. The mean change in peak expiratory flow % in the BUD group was 15.69 ± 3.59%, and in the BDP group, it was 13.59 ± 4.26% (P-value=0.04)

Conclusion

BDP and budesonide (BUD) were both found to be effective for the treatment of mild persistent asthma in children. However, we found that BUD had better efficacy compared to BDP.

## Introduction

Asthma is a common illness of childhood in which chronic inflammation of the airways are a characteristic feature. This inflammatory disorder of chronic nature requires frequent monitoring and timely intervention for preventing irreversible and long-term pulmonary function impairment. If asthma remains uncontrolled, it can significantly decrease the quality of life, especially in children through retardation of growth, exercise inability, insomnia, and frequent absenteeism from school [1].

Asthma is a chronic lung condition that really makes it hard to breathe and execute regular everyday activities. It is a disease characterized by large variations in airflow resistance in medium-sized intrapulmonary airways over short periods. The airways of asthmatic persons are found swollen, obstructed due to mucus, and compressed by adjoining muscles. If symptoms intensify and become severe within a brief period, it is considered an asthma attack. Asthma attacks can become so serious that a person needs medical treatment, or they can result in death. Individuals can experience an asthma attack if they are subjected to stimuli such as allergens, smoking, high air temperatures, and air pollution, or if they become ill due to infection. Asthma is managed with inhalers and/or oral medicine. They tend to relieve the muscles that cover the airways or reduce the swelling of the airways [2].

Various medicines are readily available, which are recommended in asthma. The basic need for inhaled corticosteroids (ICSs) lies in their anti-inflammatory mechanisms, followed by oral steroids, chromones, leukotriene modifiers, and theophylline [3]. Among them, ICSs are regarded as the most highly effective as well as the first line of treatment therapy in any level of persistent asthmatics [4]. The rates of success are based upon ICSs' ability to alleviate symptoms of asthma and therefore improve the quality of life, with fewer side effects and low bioavailability, systemically [5]. Various available ICSs include budesonide (BUD), beclomethasone dipropionate (BDP), fluticasone, flunisolide, ciclesonide, and mometasone. From them, BUD and BDP are the most widely and acceptably used especially among developing countries because of their beneficial cost-benefit ratio [6-7].

Nonetheless, it is still a matter of debate as to which of the two drugs are better for each other in terms of their efficacy. Multiple pieces of research have demonstrated the differences between the two drugs in terms of their pharmacokinetic properties. It has been observed that BDP’s affinity is higher than BUD's; however, BUD’s potency is greater. With that said, it is still not clear if these variations show any clinical significance in studies [8-15].

Overall, the advent of inhaled corticosteroid therapy has facilitated a change in the diagnosis of asthma from a predominantly symptomatic strategy (acute attack therapy) to a preventive approach that emphasizes symptomatic treatment and acute attack mitigation. Asthma serves as a risk factor in chronic airflow limitation [9]. The key component in asthma treatment is inhaled corticosteroids (ICS) [10-11].

There is a persistent increase in the prevalence of asthma despite extensive research on pathogeneses associated with asthma. Indeed, asthma is now the most widespread chronic respiratory disease in children. Asthma-related long-term respiratory problems can be avoided if timely treatment is available, particularly through the earliest possible use of anti-inflammatory therapy. Besides, due to its quick action and preferential safety, inhalation is the favored mode for asthmatic children relative to oral therapy.

BDP and BUD are drugs of choice for chronic asthma. Their invitro pharmacokinetic characteristics may vary. If any of this indicates clinically important variations in effectiveness or protection in the treatment of children with asthma remains an open question. Therefore, due to the different body lifestyles, environment, and eating choices, it would be crucial to undertake this study in the city's leading pediatric hospital for the native community, as it is unsure which drugs are more effective because there is limited information available on the same.

## Materials and methods

After approval from the institutional ethical review board (IERB) of the National Institute of Child Health (EX-13/2020), the medical records of 60 patients visiting the outpatient department OPD at the National Institute of Child Health, Karachi, were reviewed. Demographic detail (including name, age, and gender) was also obtained.

Data of the patients taking BUD 200 µg one puff twice a day and patients BDP 200 µg one puff from a metered-dose inhaler (MDI) with a valved spacer was retrieved. Spirometry was performed thrice by using a vitalograph portable spirometer that was calibrated for volume once daily with an airtight 3 l calibrated syringe with an accuracy of 15 ml. and the best values among three attempts were considered. After recording the absolute and percentage predicted values of forced expiratory volume in the first second (FEV1), patients were given the medicines for the next two months, which included random number-coded MDI and salbutamol MDI (rescue medication). The same type of valved spacers was used for the delivery of drugs from the MDI. Children were followed for two weeks. The difference between baseline and after two months of therapy was recorded on a predefined questionnaire.

Data was entered on SPSS version 21 (IBM Corp., Armonk, NY). Mean ± SD was calculated for quantitative variables like age, duration of asthma, peak expiratory flow (PEF) value at baseline, second month, and change. Frequency and percentages were computed for qualitative variables like gender. Change of peak expiratory flow was compared between both groups using independent t-test keeping. Effect modifiers like gender, duration of asthma, and age were controlled by stratification. The chi-square test was used as appropriate. P ≤0.05 was considered significant.

## Results

The demographic and clinical baseline features of the subgroups are presented and are comparable, including the spirometric baseline variable.

The mean age of children in the BUD group was 10.56+/-3.01 years, and it was 10.05+/-3.54 years in the BDP group. The mean duration of symptoms in the BUD group was 5.28 ± 3.65, and it was 4.67 ± 3.39 in the BDP group.

In the BUD group, 66.7% (n=20), and in the BDP group, 60% (n=18) children have less than 10 years of age. Gender distribution was as follows: in the BUD group, there were 56.7% (n=17) female and 43.3% (n=13) male while 50%(n=15) were female and 50% (n=15) were male in the BDP group.

**Figure 1:**
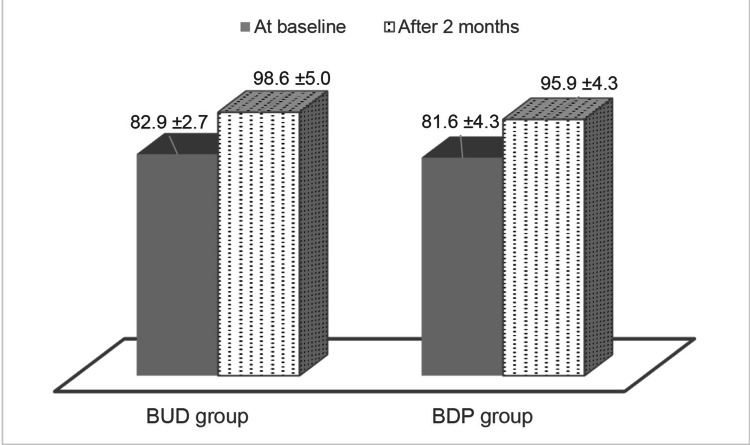
Mean peak expiratory flow at baseline and two months in both study arms BUD=budesonide, BDP=beclomethasone dipropionate

At the baseline, the mean peak expiratory ﬂow % in the BUD group was 82.9 ± 2.65% while in the BDP group, it was 81.56 ± 4.29% (P-value=0.15) (Table 1). At the end of two months, the mean peak expiratory flow percentages were 98.59 ± 4.98% and 95.87 ± 4.29%, respectively, with a P-value of 0.02. Comparing the two groups, the significant (P<0.05) improvement in peak expiratory flow (%) in the BUD group was 15.69 ± 3.59%, and in the BDP group, it was 13.59 ± 4.26% (P-value=0.04) (P-value=0.15) (Table 2). Figure 1 shows the mean peak expiratory flow at baseline and two months in both study arms.

**Table 1:** Comparison of baseline characteristics between two groups BUD=budesonide, BDP=beclomethasone dipropionate, SD=standard deviation

		BUD group	BDP group	p-value
Age (years); mean ±SD		10.6 ± 3.0	10.1 ±3.5	0.55
Age groups; n (n%)	≤10 years	20 (66.7)	18 (60)	0.59
	>10 years	10 (33.3)	12 (40)	
Gender; n (n%)	Male	13 (43.3)	15 (50)	0.60
	Female	17 (56.7)	15 (50)	
Duration of asthma symptoms (years); mean ±SD		5.3 ±3.7	4.7 ±3.4	0.51
Peak expiratory ﬂow at baseline		82.9 ±2.7	81.6 ±4.3	0.15

**Table 2:** Comparison of peak expiratory ﬂow after two-week follow up, and changes in peak expiratory flow w.r.t. age, gender, and duration of asthma symptoms *p-value < 0.05 will be considered statistically significant, BUD=budesonide, BDP=beclomethasone dipropionate

Outcome variables		BUD group mean ±SD	BDP group mean ±SD	p-value
Peak expiratory ﬂow after two months		98.6 ±5.0	95.9 ±4.3	0.02*
Change in peak expiratory ﬂow		15.7 ±3.6	13.6 ±4.3	0.04*
Age groups	≤ 10years	16.0 ±3.7	13.9 ±4.2	0.11
	> 10years	16.0 ±4.0	13.9 ±3.8	0.21
Gender	Male	15.9 ±3.6	13.8 ±3.5	0.12
	Female	16.3 ±4.1	13.7 ±4.0	0.07*
Duration of asthma symptoms	≤ 5 years	15.9 ±4.4	14.1 ±4.0	0.32
	>5 years	16.6 ±4.9	14.0 ±4.8	0.10

## Discussion

Our study reflected an improvement in the quality of life of asthmatic children, especially those receiving BUD. In the BUD group, though there was a reduction in sleep disturbance, school absenteeism, limitation of physical activities, and fewer children requiring emergency visits and rescue medication, this difference was not significant. A substantial rise in PEF was observed at the end of the first and second months in the receiving BUD group. However, daily home monitoring of PEF rate was not possible, as patients could not purchase the peak flow meter.

The findings are in favor of BUD, which is usually recommended in numerous parts of the world. These promising findings are consistent with the results of other trials assessing the success of budesonide inhalation suspension (BIS) in younger children with asthma [12-22]. A similar study in children showed an insignificant improvement with BIS treatment in 23 infants [19]. This may have been due to their patients' small sample size, fairly short treatment period, and relatively mild conditions, which did not lead to any clinical improvement. Three separate daily BIS doses (0.25 mg, 0.5 mg, 1.0 mg) were contrasted with placebo based on asthma symptom scores, using relief medicines, and morning and evening PEFs. Results showed two of the high BIS dosages showed statistically significant results. However, results were not the same once the patient leave therapy earlier. Then, the 1.0mg QD dose gives the same results as that of the placebo group or even worse.

Based on these findings, we conclude that 0.5 mg is the minimum daily dose for effective results of BIS. Several efficacy parameters (patient discontinuations, evening PEF, rescue medicines) showed that the 0.25 mg QD therapy also has significant improvements as compared to placebo. In the present study, we observed that even a low dose, i.e., 0.25 mg/d displays clinical benefits in these patients. Several other researchers studied BIS effects in children [12-20] and found that the minimum effective dose problem was not discussed explicitly since either a placebo-controlled sample was absent or a single dose (usually 1.0 mg QD or more) was used.

Although a dose-related pattern was observed in some of the effectiveness factors, generally, there was no substantial improvement differential among all the related active treatment regimens. Our results showed that regular doses of 1.0 to 2.0 mg might be required with extreme oral steroid-dependent asthma and low daily intake of 0.5 to 1.0 mg for moderately severe asthma while 0.25 mg for mild asthma may be sufficient in young children.

These 12-week study results are more comprehensive than previously reported evidence regarding the protection of inhaled glucocorticosteroids (GCS) in infants and young children (age less than eight years). The opposing reactions reported in BIS-treated patients were also not significantly different from those reported in placebo-treated patients. Furthermore, it has been observed before and after the 12-week treatment that hypothalamic-pituitary axis function was not affected by any BIS dosage; results were observed on the basis of baseline cortisol status and post-adrenocorticotropic hormone (ACTH) stimulation cortisol status.

However, active treatment and placebo groups showed similar results in those patients who showed differences in response to ACTH stimulation. As far as the safety of BIS in current work is concerned, the findings are similar to previous, related studies in children [23-25]. It should be remembered that the span of this study was just 12 weeks while long-term safety requires further studies. BIS is the first nebulizable corticosteroid formulation that has the power to manage asthma in infants and young children as a potential anti-inflammatory agent. The findings of this study indicated that BIS is active and safe for children with moderate chronic asthma at different doses and that QD dosing is an available alternative to be explored by the prescriber.

## Conclusions

In conclusion, our study showed an improvement in patients’ FEV1 treated with BUD as compared to beclomethasone administered through a spacer with a valve over a span of two months. Interestingly, we observed considerable improvement in symptoms of asthma in both groups. No significant safety concerns were identified. BUD has been found to be marginally more efficient than BDP. Both BDP and BUD can be effectively used for the treatment of asthmatic children.
